# Supplementary material for: Evaluating Differentiation Status of Mesenchymal Stem Cells by Label-Free Microscopy System and Machine Learning
Source: Cells. 2023 May 31;12(11):1524. doi: 10.3390/cells12111524 (PMC10252613; doi:10.3390/cells12111524)
Supplement: Supplementary file 1 [file cells-12-01524-s001.zip › cells-2367957-supplementary.pdf]

cells MDPI

Supporting Information for

Evaluating Differentiation Status of Mesenchymal Stem Cells by Label-Free Microscopy System and Machine Learning

Yawei Kong [1], Jianpeng Ao [2], Qiushu Chen [1], Wenhua Su [1], Yinping Zhao [3], Yiyan Fei [1], Jiong Ma [1,4,5], Minbiao Ji [2,*] and Lan Mi [1,4,*]

1 Key Laboratory of Micro and Nano Photonic Structures (Ministry of Education), Department of Optical Science and Engineering, Shanghai Engineering Research Center of Ultra-Precision Optical Manufacturing, School of Information Science and Technology, Fudan University, Shanghai 200433, China; 18110720005@fudan.edu.cn (Y.K.); 21110720091@fudan.edu.cn (Q.C.); 20110720012@fudan.edu.cn (W.S.); fyy@fudan.edu.cn (Y.F.); jiongma@fudan.edu.cn (J.M.)
2 Department of Physics, Fudan University, Shanghai 200433, China; 20110190072@fudan.edu.cn
3 Human Phenome Institute, Fudan University, Shanghai 200433, China; 19110860079@fudan.edu.cn
4 Institute of Biomedical Engineering and Technology, Academy for Engineering and Technology, Fudan University, Shanghai 200433, China
5 Shanghai Engineering Research Center of Industrial Microorganisms, The Multiscale Research Institute of Complex Systems (MRICS), School of Life Sciences, Fudan University, Shanghai 200433, China
* Correspondence: minbiaoj@fudan.edu.cn (M.J.); lanmi@fudan.edu.cn (L.M.)

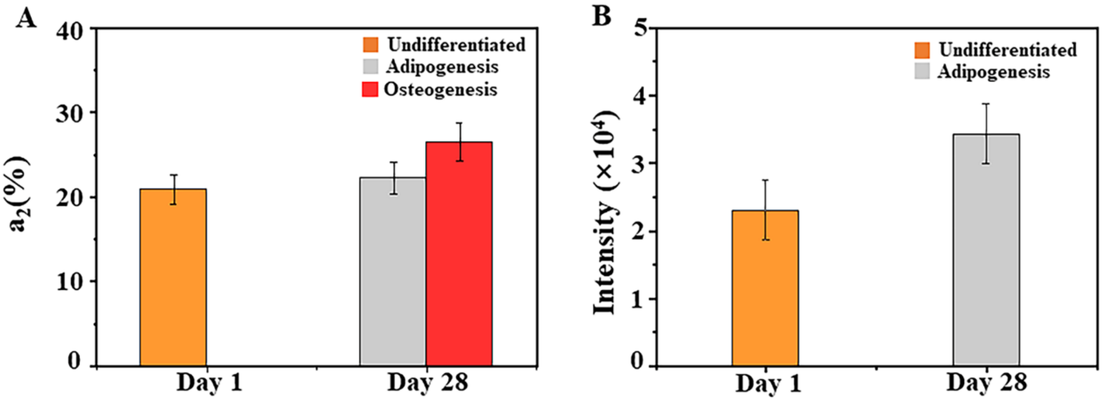

Figure S1. The changes of cells from undifferentiated to differentiated. (A) Change of NAD(P)H of cells from undifferentiated to adipogenic or osteogenesis differentiated. The percentages of protein-bound NAD(P)H (a_2) increased slightly with differentiation. (B) Change of lipid levels in differentiated cells. The lipid levels increased noticeably after 28 days of induced differentiation.

S2. Elbow rule

Usually, the number of clusters needs to be pre-specified when performing K-means++ clustering. However, due to the strong heterogeneity of cells during the induction of stem cell differentiation, as well as the complexity of metabolic states and content distribution, it is not possible to determine the number of clusters directly. The elbow method, as a method for determining the real number of clusters, provides a way to determine the

number of clusters. The core indicator is the sum of the Squared Errors (SSE) within each cluster.

$$SSE = \sum_{i=1}^{k} \sum_{e \in C_i} |e - m_i|^2 \qquad (S1)$$

where k represents the number of pre-set clusters, e denotes the elements in cluster Ci, and mi stands for the centroid of cluster Ci. SSE indicates the quality of the clustering effect. As the number of clusters k increases, the sample division becomes finer, the degree of aggregation within each cluster gradually improves, and the sum of squared errors SSE gradually decreases. When k is less than the actual number of clusters, the degree of aggregation within each cluster will increase sharply with the increase of k, so the decrease of SSE will be significant. When k reaches the actual number of clusters, the degree of aggregation within each cluster will quickly decrease as k increases, so the decrease of SSE will be reduced abruptly, and then it will tend to flatten out with the further increase of k. The relationship between SSE and k is presented as an elbow shape in the figure, and the value of k corresponding to the elbow point is the actual number of clusters in the data. The process of finding the real number of clusters based on the elbow rule is illustrated in Figure S2.

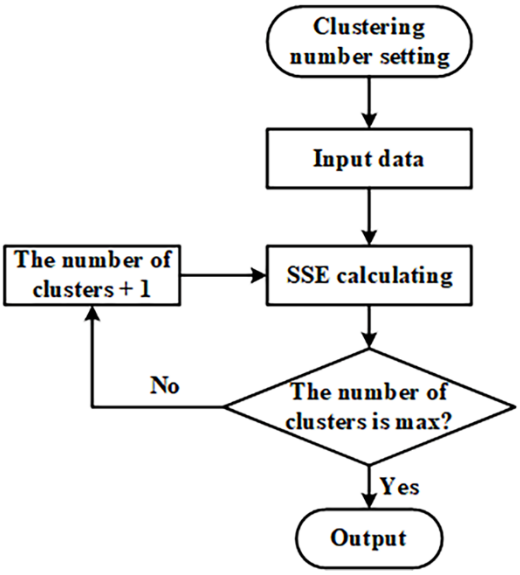

Figure S2. Elbow rule flow chart.

Figure S3 shows that when the number of clusters k is less than three, the SSE of the input feature data decreases rapidly with the increase of k, and when k is greater than three, the rate of decrease of SSE with the increase of k becomes flat for all three conditions. Therefore, the real number of classification clusters is determined as three. After determining the number of classification clusters, the K-means++ method was used to cluster the standardized feature data.

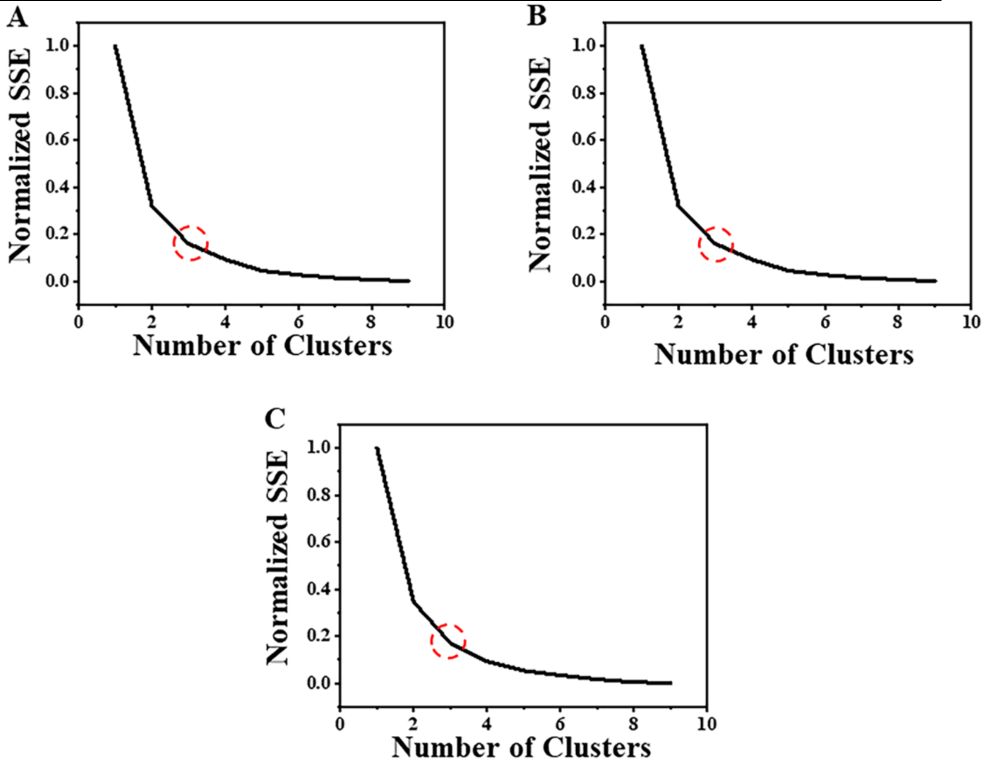

Figure S3. SSE changes with the increase number of clusters. (A) Three features of adipogenic FLIM data as input. (B) Four features of adipogenic SRS data as input. (C) Three features of osteogenic FLIM data as input.
